# Explainable time-series deep learning models for the prediction of mortality, prolonged length of stay and 30-day readmission in intensive care patients

**DOI:** 10.3389/fmed.2022.933037

**Published:** 2022-09-28

**Authors:** Yuhan Deng, Shuang Liu, Ziyao Wang, Yuxin Wang, Yong Jiang, Baohua Liu

**Affiliations:** ^1^School of Public Health, Peking University, Beijing, China; ^2^Department of Neurology, Beijing Tiantan Hospital, Capital Medical University, Beijing, China; ^3^China National Clinical Research Center for Neurological Diseases, Beijing, China

**Keywords:** intensive care unit (ICU), mortality, length of stay, readmission, prognostic prediction, deep learning

## Abstract

**Background:**

In-hospital mortality, prolonged length of stay (LOS), and 30-day readmission are common outcomes in the intensive care unit (ICU). Traditional scoring systems and machine learning models for predicting these outcomes usually ignore the characteristics of ICU data, which are time-series forms. We aimed to use time-series deep learning models with the selective combination of three widely used scoring systems to predict these outcomes.

**Materials and methods:**

A retrospective cohort study was conducted on 40,083 patients in ICU from the Medical Information Mart for Intensive Care-IV (MIMIC-IV) database. Three deep learning models, namely, recurrent neural network (RNN), gated recurrent unit (GRU), and long short-term memory (LSTM) with attention mechanisms, were trained for the prediction of in-hospital mortality, prolonged LOS, and 30-day readmission with variables collected during the initial 24 h after ICU admission or the last 24 h before discharge. The inclusion of variables was based on three widely used scoring systems, namely, APACHE II, SOFA, and SAPS II, and the predictors consisted of time-series vital signs, laboratory tests, medication, and procedures. The patients were randomly divided into a training set (80%) and a test set (20%), which were used for model development and model evaluation, respectively. The area under the receiver operating characteristic curve (AUC), sensitivity, specificity, and Brier scores were used to evaluate model performance. Variable significance was identified through attention mechanisms.

**Results:**

A total of 33 variables for 40,083 patients were enrolled for mortality and prolonged LOS prediction and 36,180 for readmission prediction. The rates of occurrence of the three outcomes were 9.74%, 27.54%, and 11.79%, respectively. In each of the three outcomes, the performance of RNN, GRU, and LSTM did not differ greatly. Mortality prediction models, prolonged LOS prediction models, and readmission prediction models achieved AUCs of 0.870 ± 0.001, 0.765 ± 0.003, and 0.635 ± 0.018, respectively. The top significant variables co-selected by the three deep learning models were Glasgow Coma Scale (GCS), age, blood urea nitrogen, and norepinephrine for mortality; GCS, invasive ventilation, and blood urea nitrogen for prolonged LOS; and blood urea nitrogen, GCS, and ethnicity for readmission.

**Conclusion:**

The prognostic prediction models established in our study achieved good performance in predicting common outcomes of patients in ICU, especially in mortality prediction. In addition, GCS and blood urea nitrogen were identified as the most important factors strongly associated with adverse ICU events.

## Introduction

Patients in the intensive care unit (ICU) are usually critically ill, presenting a high mortality risk compared with other departments in the hospital (1). In addition, readmission and prolonged length of stay (LOS) are both common clinical outcomes indicating patients’ health conditions (2, 3), critical care quality (4, 5), and medical efficiency (6). Thus, early identification of seriously ill patients and those with prolonged LOS and readmission risk and subsequent management is exceedingly important in improving patient outcomes and providing optimal allocation of medical resources.

However, traditional scoring systems, even some machine learning methods in predicting these outcomes, especially in stratifying the risk of readmission, have shown only modest results (7–10). Although part of the existing work based on machine learning models seems promising (11–13), few of them are able to take advantage of the characteristics of features collected in the ICU, which are time-series forms. Presently, these time-series problems can be approached with deep learning-based models, such as recurrent neural network (RNN) and its derived models, namely, gated recurrent unit (GRU) (14) and long short-term memory (LSTM) (15), which can learn valuable information from a large number of rapidly changing variables, making it possible to make full use of ICU data collected at a high frequency (16). Based on these advanced models, several studies have conducted prognostic prediction of patients in ICU, but most were disease-specific or ICU-specific (17–20), the clinical use of which was restricted to a specific group. To the best of our knowledge, no studies have ever predicted common outcomes while maximizing the value of these models of patients in general ICU. Furthermore, because of the complexity of these deep learning models, they are not easy to interpret, which restricts their practical application to clinical decisions (21, 22). Therefore, transparency and explainability must be considered when constructing prediction models. Recently, several methods have been introduced to improve model interpretability; among them, attention mechanisms seem to be one of the most prospective approaches (23), which have been proven to provide the foundation for clinical interpretation (24). Through explainable prediction models, significant factors can be identified at an early stage to help clinicians offer better medical interventions.

In this study, we aimed to apply three time-series deep learning models for predicting three common ICU outcomes, namely, mortality, prolonged LOS, and readmission, of patients in ICU from the Medical Information Mart for Intensive Care-IV (MIMIC-IV) database and identified predictors of high importance based on attention mechanisms to facilitate model interpretability.

## Materials and methods

### Data source and study participants

Patient information was extracted from the MIMIC-IV database (25) to conduct a retrospective cohort study. The MIMIC-IV database contains real medical records with comprehensive information for each patient, ranging from demographic information, vital signs, and laboratory tests to medication administration. All patient information was collected from those who were admitted to the emergency departments and ICU of a tertiary academic medical center in Boston, MA, United States, from 2008 to 2019. The database involves a total of 53,150 patients admitted to the ICU, and all patients’ information was de-identified.

A total of 40,083 patients were included in our study. Patients were excluded for the following reasons: (1) age ≤ 18 years or ≥ 90 years and (2) stay in the ICU for less than 24 h. In addition, we only included the first admission record if a patient was admitted to the ICU more than once, so the admission records and subject IDs corresponded.

### Predictors and outcomes

We extracted the following data from the MIMIC-IV database upon the initial 24 h of ICU admission and the last 24 h before discharge, and all of the variables were selected according to three conventional scoring systems [APACHE II (26), SOFA (27) and SAPS II (28)]: (1) basic information: age, sex, admission type, ethnicity; (2) diagnosis: AIDS, hematologic malignancy, metastatic cancer; (3) laboratory measurements: serum sodium, serum potassium, serum creatinine, hematocrit, white blood cell count, blood urea nitrogen (BUN), serum bicarbonate, bilirubin, platelets; (4) vital signs: temperature, mean arterial pressure, systolic blood pressure, heart rate, respiratory rate, PaO2, Glasgow coma score (GCS); (5) medication administration: dopamine, dobutamine, epinephrine, norepinephrine; (6) output: urinary output; (7) surgical procedures: invasive mechanical ventilation, non-invasive mechanical ventilation.

Three primary outcomes were needed for prediction in our study. One is the occurrence of death in the hospital, which was defined as whether the patient died during hospitalization, and this information can be extracted from *hospital_expire_flag* in the *admissions table* in the MIMIC-IV database. Another is the occurrence of prolonged LOS, a binary variable with a cutoff point of 75th percentile LOS of the study participants, which was 4 days in our study. Thus, patients with LOS for more than 4 days were labeled as 1, and those with LOS for less than 4 days were labeled as 0. Prolonged LOS information was calculated from the *icustays table*. The other outcome is readmission, which was defined as whether the patient was recorded as having full-cause readmission within 30 days after hospital discharge.

Data extracted from the initial 24 h after ICU admission were used to predict mortality and prolonged LOS, while data derived from the last 24 h before discharge were used to predict the risk of 30-day readmission.

### Data preprocessing and statistical analysis

Continuous variables are presented as the means ± SDs or medians and interquartile ranges and are compared using Student’s *t*-test or Wilcoxon rank-sum test according to their normality test results. Categorical variables are presented as counts and percentages and compared through the Chi-square test or Fisher’s exact test with significant *p*-values < 0.05.

According to recording frequencies, predictors can be classified into dynamic predictors and static predictors. Dynamic variables were those recorded more than once during ICU hospitalization, mostly consisting of vital signs and laboratory tests. Static variables, which included demographic information such as age, sex, and admission type, were all constant and did not change over time. The initial 24 h of ICU admission and the last 24 h before discharge were divided into a time-series of 24 steps, and all variables were obtained for each 1 h window to generate a complete dataset. For static variables, the same value of each patient was recorded 24 times. For dynamic variables, if a variable was recorded more than once in an hour, its mean value was used for aggregation, and then the last observation carried forward (LOCF) was conducted to impute missing values of time-series data. After the first missingness imputation, variables with missing rates of more than 30% were excluded. All categorical variables were one-hot encoded, so the final number of predictors was 33.

All participants were randomly split into a training set (80%) and a test set (20%). The mean value of each continuous variable in the training set was used to impute the remaining missing values in both the training set and the test set. Three deep learning models, RNN, GRU, and LSTM, were used for model development in the training set, and model performance was evaluated in terms of AUC, sensitivity, specificity, and Brier score in the test set. Variable importance according to the attention mechanism was also produced from the test set.

All data analysis procedures were conducted with SAS 9.4 and Python 3.7.

### Recurrent neural network

**FIGURE 1:**
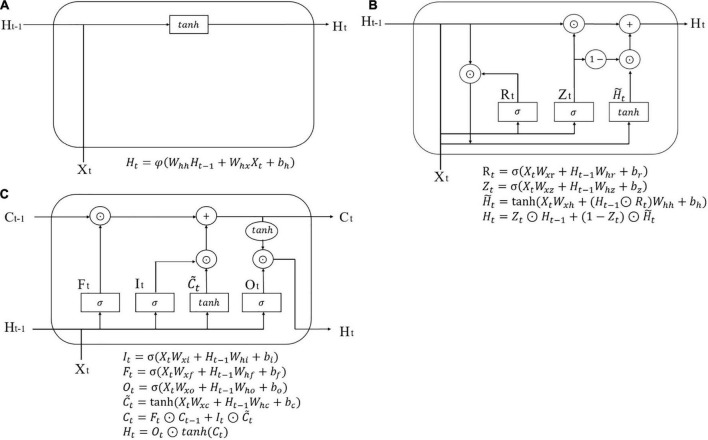
Model diagram of a single cell. **(A)** RNN; **(B)** GRU; **(C)** LSTM.

The mechanism of RNN to tackle time-series problems is that it includes a hidden layer, which incorporates information from all former steps, and with the extension of each time step, the hidden layer iteratively updates, and stores new memory. As shown in Figure 1A, *X*_*t*_ represents input variables of the present time step, while *H*_*t–1*_ is the hidden layer of the previous time step, two of which co-determine the hidden layer *H*_*t*_ of the present time step, so *H*_*t*_ contains all information of both the previous time steps and the present time step.

### Gated recurrent unit

Gated recurrent unit enriches the structure of RNN with gating systems (an update gate and a reset gate) to solve the problem of too much information kept in the hidden layer when time sequences are too long, in which the update gate (*Z*_*t*_) decides how much information to forget and how much information to keep and the reset gate (*R*_*t*_) determines how much information on former steps to forget, as shown in Figure 1B.

### Long short-term memory

Long short-term memory is more complicated than GRU. It has three gates, an input gate (*I*_*t*_), a forget gate (*F*_*t*_), and an output gate (*O*_*t*_) addition with a memory cell *C*_*t*_. The three gates are all generated by *X*_*t*_ and *X*_*t–1*_, and they separately decide how much present input information to keep, how much previous information to forget, and how much total information to output. The schematic diagram of an LSTM cell is shown in Figure 1C.

### Attention mechanism

Considering the complexity of the three deep learning models, especially LSTM, which has relatively more parameters, it would be very difficult to explain the contribution of each variable from these prediction models. Hence, an additional layer was added to each of the three models at the level of input variables; specifically, each variable of each time step (33 × 24 time-specific variables in all) was given an attention weight, which can be represented as *a*_*t*_ = *softmax*(*x*_*t*_*W*_*t*_), and the sum of the weight of each time step was equal to 1 (|*a*_*t*_| = 1), so the new input variable was represented as *X*_*new*_ = *A*⊙*X*. As a result, we ignored the possibly different contributions of each time step but focused on the contribution of each variable. Through the aggregation of all time steps, the global contribution of each variable can be generated.

## Results

### Patient characteristics

**FIGURE 2:**
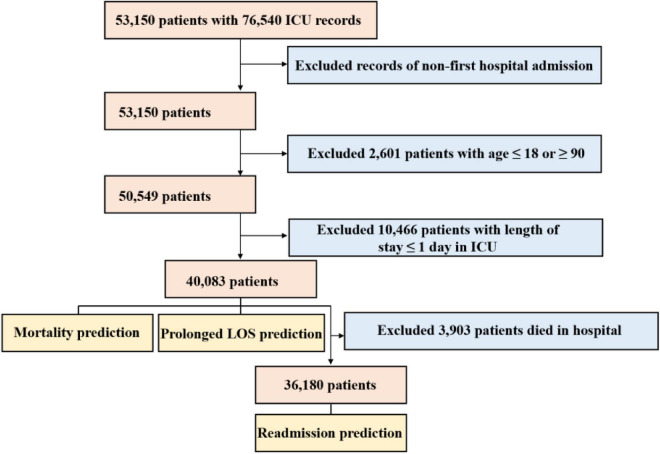
Flow chart depicting the inclusion of study participants.

A total of 40,083 patients were included in our study for the prediction of mortality and prolonged LOS after excluding those who did not meet the selection criteria, and 36,180 of them were included to predict readmission, as shown in Figure 2. Among these patients, 3,903 (9.74%) deaths occurred during hospitalization, and 11,038 (27.54%) underwent prolonged LOS. After excluding 3,903 patients who died in the hospital, 4,268 (11.79%) were readmitted to the hospital within 30 days after discharge. The comparison of basic information of these patients stratified by outcomes is shown in Table 1. Patients with in-hospital death, compared with those without, were older (*P* < 0.001), comprised more women (*P* < 0.001) and more other or unknown ethnicity (*P* < 0.001), and were more likely to be admitted to the emergency room and transferred from the hospital (*P* < 0.001), had a longer LOS in the ICU (*P* < 0.001), and were more likely to be diagnosed with metastatic cancer (*P* < 0.001) and hematologic malignancy (*P* < 0.001). Patients with prolonged LOS were also comprised of more women (*P* < 0.015) and other or unknown ethnicity (*P* < 0.001), more transferred from the hospital (*P* < 0.001), and more were diagnosed with hematologic malignancy (*P* < 0.048), while fewer were diagnosed with metastatic cancer (*P* = 0.025). Patients who were readmitted were also older (*P* < 0.001), comprised of more white people and fewer other or unknown ethnicity (*P* < 0.001), more were transferred from the hospital (*P* < 0.001) and diagnosed with metastatic cancer (*P* < 0.001) and hematologic malignancy (*P* < 0.001). The diagnosis of AIDS showed similar results between both patients with and without in-hospital death (*P* = 0.777), prolonged LOS (*P* = 0.985), and readmission (*P* = 0.146).

**TABLE 1:** Characteristics of study participants grouped by outcomes.

Characteristic	Total (*N* = 40,083)	Outcome 1			Outcome 2			Total (*N* = 36,180)	Outcome 3		
					
		Death (*N* = 3,903)	Survival (*N* = 36,180)	*P*-value	PLOS (*N* = 11,038)	Non-PLOS (*N* = 29,045)	*P*-value		Readmission (*N* = 4,268)	Non-readmission (*N* = 31,912)	*P*-value
**Age/year, Mean ± SD**	63.6 ± 16.1	68.5 ± 14.7	63.1 ± 16.2	**< 0.001**	63.7 ± 16.0	63.6 ± 16.2	0.444	63.1 ± 16.2	64.7 ± 15.4	62.9 ± 16.3	**< 0.001**
**Sex, n (%)**				**< 0.001**			**0.015**				0.412
Male	23,096 (57.6)	2,131 (54.6)	20,965 (57.9)	.	6,253 (56.6)	16,843 (58.0)		20,965 (57.9)	2,498 (58.5)	18,467 (57.9)	
Female	16,987 (42.4)	1,772 (45.4)	15,215 (42.1)	.	4,785 (43.4)	12,202 (42.0)		15,215 (42.1)	1,770 (41.5)	13,445 (42.1)	
**Ethnicity, n (%)**				**< 0.001**			**< 0.001**				**< 0.001**
White	26,768 (66.8)	2,307 (59.1)	24,461 (67.6)	.	7,044 (63.8)	19,724 (67.9)		24,461 (67.6)	2,998 (70.2)	21,463 (67.3)	
Black American	3,540 (8.8)	289 (7.4)	3,251 (9.0)	.	934 (8.5)	2,606 (9.0)		3,251 (9.0)	394 (9.2)	2,857 (9.0)	
Asian	1,178 (2.9)	116 (3.0)	1,062 (2.9)		291 (2.6)	887 (3.1)		1,062 (2.9)	125 (2.9)	937 (2.9)	
Hispanic	1,423 (3.6)	103 (2.6)	1,320 (3.6)		373 (3.4)	1,050 (3.6)		1,320 (3.6)	138 (3.2)	1,182 (3.7)	
Others/Unknown	7,174 (17.9)	1,088 (27.9)	6,086 (16.8)		2,396 (21.7)	4,778 (16.5)		6,086 (16.8)	613 (14.4)	5,473 (17.2)	
**Admission location, n (%)**				**< 0.001**			**< 0.001**				**< 0.001**
Emergency room	17,587 (43.9)	2,024 (51.9)	15,563 (43.0)	.	4,862 (44.0)	12,725 (43.8)		15,563 (43.0)	1,915 (44.9)	13,648 (42.8)	
Physician referral	10,154 (25.3)	412 (10.6)	9,742 (26.9)		2,073 (18.8)	8,081 (27.8)		9,742 (26.9)	870 (20.4)	8,872 (27.8)	
Transfer from hospital	9,946 (24.8)	1,236 (31.7)	8,710 (24.1)	.	3,511 (31.8)	6,435 (22.2)		8,710 (24.1)	1,213 (28.4)	7,497 (23.5)	
Others	2,396 (6.0)	231 (5.9)	2,165 (6.0)		592 (5.4)	1,804 (6.2)		2,165 (6.0)	270 (6.3)	1,895 (5.9)	
**LOS/day, Mean ± SD**	4.1 ± 5.3	6.2 ± 6.8	3.9 ± 5.0	**< 0.001**	9.6 ± 7.6	2.0 ± 0.8	**< 0.001**	3.9 ± 5.0	5.3 ± 6.9	3.7 ± 4.7	**< 0.001**
**Metastatic cancer, n (%)**				**< 0.001**			**0.025**				**< 0.001**
Yes	4,715 (11.8)	776 (19.9)	3,939 (10.9)		1,234 (11.2)	3,481 (12.0)		3,939 (10.9)	552 (12.9)	3,387 (10.6)	
No	35,368 (88.2)	3,127 (80.1)	32,241 (89.1)	.	9,804 (88.8)	25,564 (88.0)		32,241 (89.1)	3,716 (87.1)	28,525 (89.4)	
**Hematologic malignancy, n (%)**				**< 0.001**			**0.048**				**< 0.001**
Yes	1,278 (3.2)	257 (6.6)	1,021 (2.8)		383 (3.5)	895 (3.1)		1,021 (2.8)	165 (3.9)	856 (2.7)	
No	38,805 (96.8)	3,646 (93.4)	35,159 (97.2)	.	10,655 (96.5)	28,150 (96.9)		35,159 (97.2)	4,103 (96.1)	31,056 (97.3)	
**AIDS, n (%)**				0.777			0.985				0.146
Yes	47 (0.1)	4 (0.1)	43 (0.1)		13 (0.1)	34 (0.1)		43 (0.1)	2 (0.0)	41 (0.1)	
No	40,036 (99.9)	3,899 (99.9)	36,137 (99.9)	.	11,025 (99.9)	29,011 (99.9)		36,137 (99.9)	4,266 (100)	31,871 (99.9)	

PLOS, prolonged length of stay; non-PLOS, non-prolonged length of stay; AIDS, acquired immune deficiency syndrome. The bold font designates the statistically significant variables with *p* value less than 0.05.

### Model performance

**FIGURE 3:**
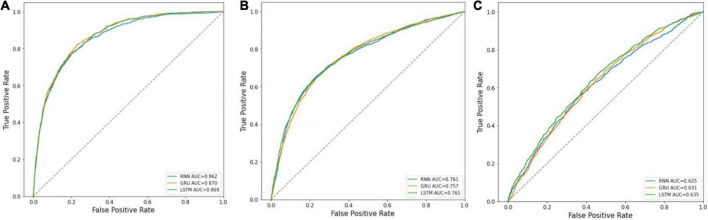
ROC curves of RNN, GRU, and LSTM. **(A)** Mortality prediction; **(B)** prolonged LOS prediction; **(C)** 30-day readmission prediction.

The receiver operating characteristic (ROC) curves of the three prediction models in predicting in-hospital mortality, prolonged LOS, and 30-day readmission are shown in Figures 3A–C. The AUCs of RNN, GRU, and LSTM in predicting mortality were 0.862 ± 0.001, 0.870 ± 0.001, and 0.869 ± 0.002, respectively, and those in prolonged LOS prediction were 0.761 ± 0.002, 0.757 ± 0.011, and 0.765 ± 0.003, respectively. The AUCs of readmission prediction reached only 0.625 ± 0.008, 0.631 ± 0.011, and 0.635 ± 0.018 for the three deep learning models. Other performance metrics, namely, sensitivity, specificity, and Brier score, are shown in Table 2.

**TABLE 2:** Model performance in predicting hospital mortality, PLOS, and 30-day readmission of patients in ICU.

Performance	Mortality prediction			PLOS prediction			30-day readmission prediction		
			
	RNN	GRU	LSTM	RNN	GRU	LSTM	RNN	GRU	LSTM
AUC	0.862 ± 0.001	**0.870 ± 0.001**	0.869 ± 0.002	0.761 ± 0.002	0.757 ± 0.011	**0.765 ± 0.003**	0.625 ± 0.008	0.631 ± 0.011	**0.635 ± 0.018**
Sensitivity	0.787 ± 0.012	**0.796 ± 0.015**	0.790 ± 0.020	0.651 ± 0.009	**0.666 ± 0.018**	0.655 ± 0.027	0.658 ± 0.036	0.652 ± 0.083	**0.691 ± 0.064**
Specificity	**0.786 ± 0.011**	0.782 ± 0.012	0.783 ± 0.017	**0.771 ± 0.009**	0.741 ± 0.012	0.760 ± 0.024	**0.567 ± 0.039**	0.541 ± 0.072	0.524 ± 0.061
Brier Score	**0.073 ± 0.003**	0.087 ± 0.006	0.082 ± 0.010	**0.169 ± 0.006**	0.204 ± 0.019	0.185 ± 0.014	0.105 ± 0.001	0.105 ± 0.002	**0.104 ± 0.009**

AUC, area under the curve; PLOS, prolonged length of stay; RNN, recurrent neural network; GRU, gated recurrent unit; LSTM, long short-term memory. The bold font represents the best score of the three models.

### Variable significance

**FIGURE 4:**
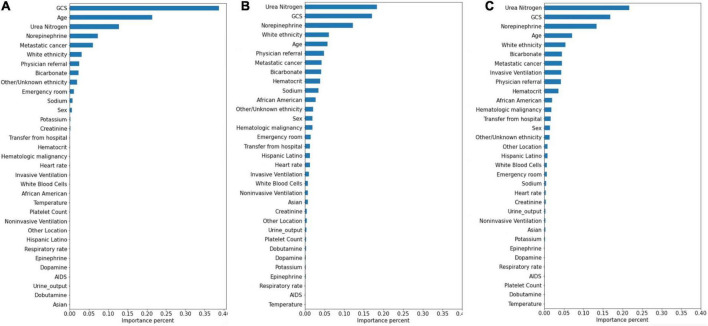
Variable importance generated by mortality prediction models. **(A)** RNN; **(B)** GRU; **(C)** LSTM.

**FIGURE 5:**
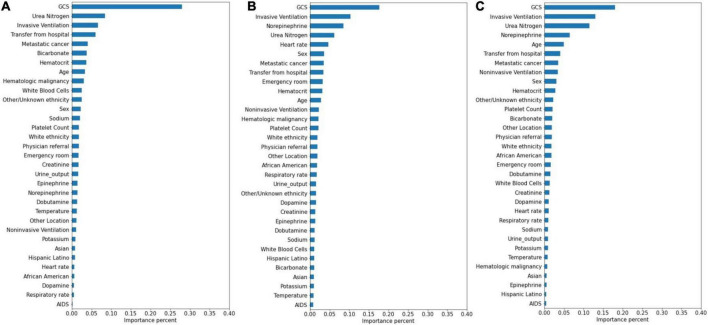
Variable importance generated by prolonged LOS prediction models. **(A)** RNN; **(B)** GRU; **(C)** LSTM.

**FIGURE 6:**
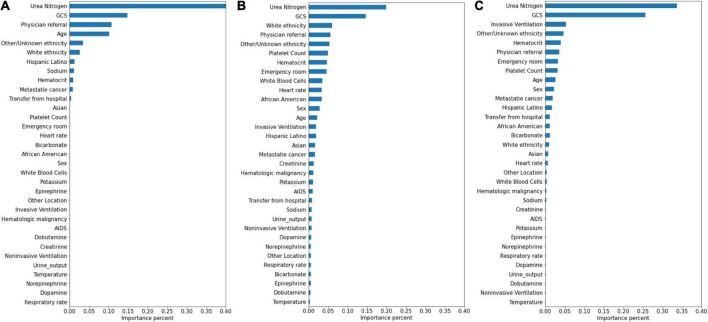
Variable importance generated by 30-day readmission prediction models. **(A)** RNN; **(B)** GRU; **(C)** LSTM.

The significance of the variables is shown in Figures 4–6. All three prediction models (RNN, GRU, and LSTM) indicated the important roles of GCS, age, blood urea nitrogen, and administration of norepinephrine in predicting mortality. GCS, invasive ventilation, and blood urea nitrogen were all among the top five significant predictors for prolonged LOS prediction. Blood urea nitrogen, GCS score, and ethnicity were strong predictors for 30-day readmission prediction.

## Discussion

In this study, three time-series deep learning models were applied to predict in-hospital mortality, prolonged LOS, and 30-day readmission with conventional and easily available variables in ICU settings, and influential factors associated with the three outcomes were identified through attention mechanisms to enhance model interpretability.

Our study focused on the outcome prediction of general patients without distinguishing their diseases, and the results showed in-hospital mortality of 9.74%, a prolonged LOS of 27.54%, and 30-day readmission of 11.79%, which were roughly consistent with previous studies (29, 30). For better practical use in clinical settings, we only included variables that are commonly used and easily available according to three traditional scoring systems [APACHE II (26), SOFA (27), and SAPS II (28)] and collected within 24 h, so compared with other similar studies, the number of variables in this study was relatively small, which partly explained the not very outstanding performance of our prediction models. For example, in Golas’s study, 3,512 variables were included (31) and in Sherman’s study, 165 variables were included (32), while in our study, only 33 variables were included, which were all among the common clinical measurement indicators. Specifically, the values of AUC indicated good discrimination capability in mortality prediction, moderate in prolonged LOS, and not as good in readmission. All prediction models were trained with a 24-h time window, which was a comprehensive consideration of various conditions, such as the significance of each period, the complexity of variable availability, and the missingness rate. Nevertheless, the length of the time window can also have a certain impact on model performance. In Na’s study, the best-performing model (GRU) was trained with 8/16/24/48-h time windows, and the overall tendency indicated that the extended time window corresponded to better predictive performance (33). In addition, the performance of the readmission prediction model may be strongly affected by the period of readmission, ranging from 24 h to 30 days in existing studies (34–36); usually, the shorter the time interval is the better the prediction capability. Thus, using a relatively narrow time window, which is 24 h, to predict long-term outcomes theoretically resulted in a weak predictive capability. However, the result is still competitive in all three outcome predictions because of the application of deep learning models with a small quantity of time-series variables (8, 9, 31, 37).

The results of the performances of the three deep learning modes (RNN, GRU, and LSTM) did not differ greatly in predicting outcomes, and this was inconsistent with what was obtained by Na’s study (33). For a similar task (mortality prediction using RNN, GRU, and LSTM with variables collected within a 48-h observation window), GRU and LSTM performed better than RNN. In their study, the observation window was double-length, which may be related to the difference in the results. The superiority of LSTM and GRU is that their additional gate systems can better select important information stored in hidden layers on each time step, so when the time window is too short, the information contained is more likely to be undiscardable so that the advantages of LSTM and GRU cannot be reflected (23).

Attention mechanisms allowed us to identify important features used by three different models in prediction, and the influential variables of each outcome selected by different deep learning models also did not differ greatly. The GCS was identified as the top important factor for mortality, prolonged LOS, and readmission prediction, and the same results can also be extracted from other similar studies. For example, some studies have concluded that GCS is an independent mortality-related factor and has the most significant feature importance in some specific diseases (38, 39). This variable was also demonstrated to be one of the most important determinants of prolonged LOS in patients with traumatic brain injury (40). Moreover, in Oh’s study, 2.28-fold higher unplanned 2-day readmissions were associated with GCS scores less than 13 (41). A lower GCS score indicates more severely impaired consciousness, which may lead to a poor outcome if timely medical intervention is not conducted (42). Age was also demonstrated to have a strong relationship with in-hospital mortality in the ICU by previous studies (43, 44), with a higher mortality rate occurring among elderly patients. These patients generally have reduced immunity, underlying chronic diseases, and worse recovery ability, which may complicate their health status and result in adverse outcomes (45, 46). In Martin’s study, BUN was discovered to have a significant association with 28-day mortality (47), and Jamshid’s study identified BUN as one of the factors with the highest predictive values to predict the risk of mortality from patients with severe COVID-19 (48), which also provides support for our results. BUN was also identified as a significant variable for prolonged LOS and readmission prediction, and the same results can also be found in homogeneous studies (49, 50). The increased level of BUN is associated with kidney damage, which is supported by multiple mechanisms (51). We also included some medication administration information following SOFA scoring systems (27), and the results showed that norepinephrine, which was recommended as first-line therapy for cardiogenic shock (52), had decisive implications on mortality prediction. This result was also generated by Lu’s study, which concluded that patients in cardiogenic shock treated with norepinephrine had significantly increased short-term mortality rates (53). These patients, especially those in refractory shock, usually had an extremely poor prognosis, which lead to higher mortality (54). We also found that invasive ventilation was a decisive predictor for prolonged LOS, a risk factor also suggested by a meta-analysis containing 28 articles (3). In the prediction of readmission, the results showed that ethnicity was a decisive predictor, with the white people owning an increased probability for readmission and other/unknown ethnicity decreasing. In Mukhopadhyay’s study, the results also showed that ethnicity was independently associated with hospital readmissions (55).

There are several limitations to our study. First, we excluded some variables that may have predictive values because of high missingness rates, such as the mean arterial blood pressure and bilirubin, and the insurance variable, which may influence LOS, was also not included considering that more than half of the insurance type was labeled “Others.” Second, as a single-center study, the generalizability and representation of our conclusion still need to be demonstrated by other data sources. Third, the alternative variables may still be not comprehensive. For example, the diagnosis at ICU admission was not considered a predictor in our study, which may affect the application and generalization of this model in different patient groups. More variables that are easily available need to be explored to further improve model performance.

## Conclusion

Three time-series deep learning models were applied for the prediction of three common ICU outcomes, namely, mortality, prolonged LOS, and readmission. The prediction models reached good performance, especially in mortality prediction, which is of great value in clinical settings considering the conventional and easily available variables incorporated. Our results also indicate that GCS and blood urea nitrogen were highly associated with adverse outcomes of patients in ICU, and focusing on these variables can better assist clinical decisions.

## Data availability statement

All data analyzed in this study were obtained from the MIMIC-IV database, which can be found at https://physionet.org/about/database/.

## Ethics statement

Ethical review and informed consent were not required for this study as the study database, the MIMIC-IV database is publicly available, and all patient data are de-identified.

## Author contributions

YD designed the study and wrote the manuscript draft. BL and YJ critically revised the manuscript. SL assisted with the study protocol and data analysis. ZW contributed to manuscript editing and model explanation. YW helped with manuscript revision. All authors read and approved the final manuscript.
